# Exploring faculty experiences and perceptions of interprofessional co-debriefing practice in healthcare simulation: a qualitative study protocol

**DOI:** 10.1136/bmjopen-2025-109231

**Published:** 2025-10-21

**Authors:** Prashant Kumar, Olivia Groom, Stephen Paterson, Neil McGowan, Russell Allan, Kathryn Sharp, Susan Somerville

**Affiliations:** 1 Department of Medical Education, NHS Greater Glasgow and Clyde, Glasgow, Scotland, UK; 2 School of Medicine, Dentistry and Nursing, University of Glasgow, Glasgow, Scotland, UK; 3 Department of Anaesthesia, NHS Greater Glasgow and Clyde, Glasgow, Scotland, UK; 4 School of Medicine, University of Dundee, Dundee, Scotland, UK; 5 Department of Medicine, Royal Alexandra Hospital, Paisley, Scotland, UK; 6 Department of Critical Care, Queen Elizabeth University Hospital, Glasgow, Scotland, UK; 7 School of Health and Life Sciences, Glasgow Caledonian University, Glasgow, Scotland, UK; 8 Royal Hospital for Children and Young People, NHS Lothian, Edinburgh, Scotland, UK; 9 Centre for Medical Education and Dundee Institute for Healthcare Simulation, University of Dundee, Dundee, Scotland, UK

**Keywords:** Interprofessional Relations, MEDICAL EDUCATION & TRAINING, EDUCATION & TRAINING (see Medical Education & Training), QUALITATIVE RESEARCH

## Abstract

**Abstract:**

**Introduction:**

Interprofessional co-debriefing, whereby facilitators from different healthcare professional backgrounds jointly facilitate debriefings, is increasingly common in simulation-based education. This approach can enhance learning by incorporating diverse perspectives and distributing cognitive workload, but it may also expose tensions linked to professional identity, hierarchy and power dynamics between debriefers. While learner outcomes and debriefing strategies in general are well studied, little is known about faculty experiences of interprofessional co-debriefing or how sociocultural factors influence this practice. Addressing this gap is crucial to optimise faculty development and support effective interprofessional education. This study will qualitatively explore the experiences and perceptions of simulation educators engaged in interprofessional co-debriefing, with a focus on the influence of sociocultural factors on their practice.

**Methods and analysis:**

This UK-based qualitative study will recruit up to 30 healthcare simulation educators with experience of interprofessional co-debriefing. Participants will be purposively sampled from simulation networks, centres and academic institutions, with snowball sampling to broaden reach. Semistructured interviews will be conducted online via Microsoft Teams, guided by a topic framework developed by the research team. Interviews will be audio-recorded, transcribed verbatim and anonymised. Underpinned by constructivist and constructionist paradigms, data will be analysed using reflexive thematic analysis following Braun and Clarke’s six-phase approach. Three researchers will independently code transcripts, with themes refined through iterative team discussions to ensure rigour and transparency.

**Ethics and dissemination:**

Ethical approval has been granted by the University of Glasgow School of Medical and Life Sciences Ethics Committee (Ref No: 200240285). All participants will provide informed written consent, and data will be handled in accordance with data protection regulations. Findings will be disseminated via peer-reviewed publications, conference presentations and professional networks, with a summary provided to participants. This study will offer novel insights into the underexplored area of interprofessional co-debriefing, specifically how sociocultural dynamics may influence and shape practice, potentially informing faculty development and best practice moving forward.

**STRENGTHS AND LIMITATIONS OF THIS STUDY**

- This is a theoretically grounded qualitative study that, through a rigorous methodology, will explore faculty experiences and perceptions of interprofessional co-debriefing in healthcare simulation, with a focus on how professional identity, hierarchy and power imbalances influence debriefers.
- A semistructured interview format ensures a replicable methodology which retains flexibility to allow us to explore novel and complex concepts in depth and thus gain nuanced insights and explanations that may help inform future best practice.
- Our data will be analysed via reflexive thematic analysis, a rigorous process well-matched to the depth, complexity and contextual richness of data. Furthermore, this process explicitly recognises the active interpretative role that we, as researchers, play in the co-construction of meaning and knowledge.
- The study will only recruit UK-based simulation educators, thereby potentially limiting the transferability of the findings.
- While we seek to recruit simulation educators from a range of varied healthcare backgrounds, the recruitment strategy cannot guarantee this will occur, thus potentially leading to overrepresentation of particular views and perspectives.

## Introduction

 Simulation-based education (SBE) is a widely adopted educational technique practised across varying contexts within healthcare professions education.^1^ Ranging from table-top exercises and clinical skills training to fully immersive scenarios, it is a technique that creates a controlled environment that allows participants to experience a representation of real clinical situations for the purpose of learning, reflection and practice.^2^ There is growing evidence that SBE improves both clinical knowledge retention and improvements in both individual and team-based sociocognitive and behavioural skills.^3^,^8^

SBE is increasingly used in the context of interprofessional education,^18^,^13^ where participants from different professional backgrounds learn with, from and about each other to strengthen collaborative practice.^14 15^ Beyond fostering teamwork, interprofessional SBE, through post-scenario debriefing, also offers a rare opportunity to explore entrenched socio-historical dynamics such as power imbalances, status, hierarchies and professional divisions, and to challenge assumptions that may otherwise remain unspoken.^1 10^

Debriefing is defined as ‘discussion between two or more individuals in which aspects of a performance are explored and analysed with the aim of gaining insight that impacts the quality of future clinical practice’.^16^ Research literature has highlighted the critical role of debriefing during SBE, in deepening learning and promoting reflective practice.^16^,^19^ The presence of a skilled and experienced facilitator to help guide learners through this reflective learning process has commonly been described as one of, if not the, most important element of debriefing practice,^1720^,^22^ although this assumption is increasingly challenged.^23^ While many simulation educators practise as a sole facilitator during debriefings, the practice of co-debriefing, where more than one facilitator is present, is becoming increasingly common.^24^

Co-debriefing can enrich learning for both faculty and learners by bringing diverse perspectives, complementary facilitation styles and shared experience, while also reducing individual cognitive load and offering real-time mentorship and support for less experienced debriefers.^24^,^26^ However, introducing multiple debriefers can also create friction, including discordance, personality clashes, perceived power struggles and competing agendas.^24 25^ Particularly during interprofessional SBE, simulation educators may choose to engage in interprofessional co-debriefing, whereby facilitators who hail from different professional backgrounds co-debrief together.^24 25^ In such settings, due to the increased complexity and sociocultural dynamics of both the learner group and the faculty, both the benefits and challenges of co-debriefing can be heightened.^24 25^ Historically embedded sociocultural issues of status, power imbalances, hierarchy and professional divisions amongst debriefers may make co-debriefing in interprofessional contexts much more challenging, but they also create unique opportunities to surface assumptions, foster critical reflection and model authentic interprofessional teamwork.^9 10 25 26^

Despite its growing prevalence within interprofessional SBE, interprofessional co-debriefing remains underexplored in the empirical literature.^25^ Existing studies have only touched on aspects of learner and faculty experiences within broader investigations,^9 27^ or examined power imbalances between debriefers and learners following interprofessional simulation.^28^ However, there is little focused examination of the faculty experience and their perceptions, particularly how entrenched sociocultural factors such as professional identity, hierarchy and power imbalances influence and shape co-debriefing practice. Understanding these influences is critical as they can subtly facilitate or hinder collaboration, impact the learning environment and ultimately affect the quality of interprofessional education. By addressing this gap, this study will generate insights that may inform faculty development, optimise debriefing practices and strengthen the educational value of interprofessional simulation.

### Theoretical lens

This study will be conducted through constructivist and constructionist theoretical lenses. Constructivism purports that individuals socially construct impressions and schemas that help foster deeply personal meanings from subjective experiences.^29 30^ Constructionism champions the profound influence that cultural norms and contextual factors have on determining how individuals construct this personal meaning from such subjective experiences.^30 31^ These theoretical underpinnings will allow us to challenge the concept of ‘one objective reality’ and instead explore the existence of multiple subjective realities constructed by individuals and groups, even when derived from the same nominal experience.^32^ Such theories are especially pertinent when examining complex and dynamic sociocultural concepts such as those experienced during interprofessional co-debriefing.

### Aim and research questions

This study aims to explore faculty experiences and perceptions of interprofessional co-debriefing in healthcare simulation. Specifically, the study aims to address the following questions:

1. What are the experiences and perceptions of faculty who engage in interprofessional co-debriefing practice?
2. How does professional identity influence their practice in interprofessional co-debriefing contexts?
3. How do faculty experience and perceive hierarchy and power imbalances in the context of interprofessional co-debriefing?

## Methods and analysis

### Study design

This qualitative study will examine faculty experiences and perceptions of interprofessional co-debriefing in healthcare simulation, focussing on how professional identity, hierarchy and power imbalances influence debriefers and shape their practice. Data will be collected using semistructured interviews conducted online via the Microsoft Teams platform. Interviews will last approximately 30–45 min and will be completed between April and October 2025. A total number of up to 30 interviews is anticipated: a pragmatic maximum to ensure data richness and depth while permitting broad coverage across professional groups, thereby safeguarding diversity in background and perspectives within the sample. This is aligned with the concept of information power, a model used to guide adequate sample sizes for qualitative studies, whereby the size of the sample is dependent on (1) the study aims, (2) the specificity and heterogeneity of recruited participants, (3) the extent to which existing theory informs analysis, (4) the quality and richness of interview dialogue and (5) the analytical strategy.^33^ If sufficient information power is attained prior to reaching the maximum number of interviews, further interviews will cease. The decision process governing such judgements will be clearly documented in the study audit trail. Study participants will be interviewed using a predefined topic guide (online supplemental file 1). This topic guide is informed by our research aims and has been iteratively developed through discussion among the research team. It provides structured and comprehensive guidance, while allowing sufficient scope to explore novel, unanticipated or complex matters as appropriate.^34^ As a research team, we will undertake individual reflexive memos and group debrief discussions to monitor whether questions and prompts inadvertently prime responses and will engage in the process of continuous iterative revision and refinement of the topic guide to maintain open exploration of the relevant topics. Semistructured interviews involve a participant answering a combination of prepared and open questions with subsequent participant-led discussion. This enables focused, deep exploration into the participant’s experiences of the research topic, as well as well as incorporating flexibility to highlight novel and nuanced findings.^35 36^ This makes semistructured interviews a flexible and powerful method of data collection in qualitative research, whereby it is still possible to pursue unanticipated, but relevant, areas of interest that arise during the conversation.^34 36^ This approach was chosen over other qualitative methods such as focus groups, so as to minimise the impact of interprofessional dynamics that we are aiming to characterise with the data collected, with the intention of increasing participant candour.^37^ We acknowledge that the tone and rapport fostered between interviewer and participant helps shape the interview and can facilitate more meaningful and comprehensive discussion, therefore allowing for rich data generation.^34 36 38^

### Participants: recruitment and sampling strategy

Inclusion criteria are that participants:

1. Are over 18 years old.
2. Are professionally based in the UK.
3. Are qualified healthcare professionals of any clinical background.
4. Have experience, as faculty, of interprofessional co-debriefing.

Participants will be sampled from UK-based simulation associations, simulation centres, networks and academic institutions via purposive sampling. This strategy will allow us to actively and iteratively seek diversity in perspectives by employing targeted outreach to under-represented professions, backgrounds, experience levels and geographical regions where needed, as the study progresses.^39^ Prospective participants will be emailed a written invitation alongside a participant information sheet and privacy statement (onlinesupplemental files 2^3^) and invited to contact the research lead if they would like further information or wish to enrol in the study. Snowball sampling from these participants will also be used to recruit additional colleagues who meet the above inclusion criteria and are willing to participate in the study. We acknowledge that the findings from this study will be UK-centred. As such, the sociocultural dynamics of interprofessional co-debriefing within this context, specifically in respect to professional status, hierarchy and power, may not be directly transferrable to other international settings, where different sociocultural norms and simulation practices exist. Our aim, therefore, is not to afford judgement on transferability, but to transparently provide sufficiently detailed descriptions of context and analytical interpretations, such that readers can form their own judgements regarding transferability to their local settings.

### Consent

Written informed consent will be collected, with participants being asked to sign and return an electronic consent form prior to proceeding to interview (online supplemental file 4). This includes recognition of the limits of confidentiality outlined in the participant information sheet (online supplemental file 2). Enrolment in the study will be completely voluntary, with opportunity to withdraw without penalty. Participants will be informed that data can only be erased, and therefore not included in the data analysis, prior to transcription and anonymisation. Once the interviews have been transcribed and anonymised, data will be included in the analysis. Participants will be informed that they are free to skip questions or refuse to answer questions if they so wish and that they do not need to provide any reasoning as to why they may wish to do so. Consent will be reconfirmed verbally at the time of interview.

### Data collection and management

Interviews will be video recorded and transcribed on the online platform Microsoft Teams. These video recordings and the subsequent transcripts will be stored on an encrypted and password-protected folder on Microsoft OneDrive for Business, operated securely by the *University of Glasgow*. Only members of the research team will have access to this folder. Video recordings will be securely deleted once transcriptions have been independently verified for accuracy by a member of the research team. We aim to have transcriptions confirmed for accuracy within 1 week of interview. The anonymised transcripts will be kept in this folder for a period of 10 years in accordance with the *University of Glasgow’s* data protection policies and regulations. At this point, the data will be securely deleted. Participant names, their completed consent forms and contact details will also be securely stored on this drive, in a separate encrypted and password-protected folder, for the same time frame. It will therefore not be possible to link and identify the names, contacts or consent forms with the anonymised transcription files. All subsequent transcripts, data analysis, coding templates, tables, figures and reports will be stored on this same channel. Access to all anonymised data will be restricted to the study team only and will not be available for secondary use by other researchers.

All participants will be clearly informed of these intentions, both via the written participant information sheet (online supplemental file 2) and as part of the consenting process prior to the interview being conducted. We have determined there to be a low risk of loss of personal data. Via the privacy notice (online supplemental file 3), participants will be informed of how to contact the data protection officer at the *University of Glasgow* should they have any concerns regarding how their data has been handled.

### Data analysis

Data will be analysed by reflexive thematic analysis (RTA) according to Braun and Clarke’s framework,^40^ by three researchers (PK, SP and OG). The framework comprises the following six stages^40^:

1. Familiarising yourself with the data set.
2. Coding.
3. Generating initial themes.
4. Developing and reviewing themes.
5. Refining, defining and naming themes.
6. Writing up.

In this form of qualitative analysis, researchers, through their reflexive interpretative analysis of the patterns of data and their meanings, have an active and central role in knowledge generation, and as such may resist notions of positivistic data interpretation.^40 41^ Themes do not simply surface from the data; rather, they are fashioned by the researcher(s) as they analyse, compare and contrast the codes.^40^ Embracing the subjectivity and reflexivity that this research process entails, we will ensure transparency in how we engage in the data analysis, such that readers can judge how our experiences and perspectives as interprofessional simulation educators who engage in interprofessional co-debriefing may influence the evaluation and interpretation of the data.^42^ As per Braun and Clarke’s framework,^40^ we will familiarise ourselves with the content of the transcripts and generate initial codes for any key features across the data set. These initial codes will then be grouped into broader themes, which will then be reviewed against the full data set to ensure all significant findings are incorporated. Each theme will then be refined to ensure they are clearly named and defined. Any differences or discrepancies in the analysis will be discussed and addressed to reach a consensus. Importantly, rather than a linear process, RTA is recursive in nature, in which we expect to move back and forth between the different stages of the analysis as our insights and understanding develop.^40^ Adhering to the COREQ (Consolidated Criteria for Reporting Qualitative Research) guidelines,^42^ the results of the RTA will then be compiled into a manuscript which we aim to publish in a peer-reviewed scientific journal and disseminate to the healthcare simulation community.

### Reflexivity

Consistent with a constructivist paradigm, our analysis will explicitly acknowledge how the perspectives, experiences and values of the research team interact with the data to shape interpretation. Our research group is a collective of simulation educators, practitioners and academics who all have extensive experience of interprofessional co-debriefing in healthcare simulation. Furthermore, we represent a range of clinical professional backgrounds, including medicine (PK, OG, RA, NM), nursing (SP, SS) and physiotherapy (KS). PK, OG, SP, RA and NM are active clinically. PK, SP, KS and SS have experience of conducting qualitative research, while PK, KS and SS are experienced in supervising other qualitative research projects related to healthcare simulation practice. PK is the lead researcher for this study and SS is the overall project supervisor. Five researchers will conduct the interviews (PK, SP, KS, NM, SS). There is the possibility that study participants may be known contacts of the interviewing team. This possibility will be clearly articulated through the recruitment process. As a collective research team, we declare no conflicts of interest. This protocol has been developed, peer-reviewed and published in order to optimise transparency and thus enhance trustworthiness and reduce potential bias.^43^

### Patient and public involvement

Due to the research topic, there was no involvement of patients or the public in the design of the study. However, findings will be disseminated to study participants on completion.

### Ethics and dissemination

Ethical approval for this study has been obtained from *University of Glasgow* School of Medical and Life Sciences Ethics Committee (Ref No. 200240285). Study participants will not be incentivised, either monetarily or otherwise, to participate in this study, with their involvement being entirely voluntary. This will be clearly communicated with all potential study participants during the recruitment and consent process. Ethical principles and standards detailed in the Declaration of Helsinki^44^ will be adhered to throughout the entirety of the study duration, as detailed in the methods section of this protocol.

The study findings will be disseminated via presentations at relevant scientific meetings and conferences, and publication of findings in an appropriate peer-reviewed academic journal. We will also use professional social media platforms (X, BlueSky, LinkedIn) to broaden the reach of our findings and engage the wider healthcare simulation community.

## Supplementary material

10.1136/bmjopen-2025-109231 online supplemental file 1

10.1136/bmjopen-2025-109231 online supplemental file 2

10.1136/bmjopen-2025-109231 online supplemental file 3

10.1136/bmjopen-2025-109231 online supplemental file 4
