# Supplementary material for: Exploring faculty experiences and perceptions of interprofessional co-debriefing practice in healthcare simulation: a qualitative study protocol
Source: BMJ Open. 2025 Oct 21;15(10):e109231. doi: 10.1136/bmjopen-2025-109231 (PMC12548587; doi:10.1136/bmjopen-2025-109231)
Supplement: online supplemental file 1 [file bmjopen-15-10-s001.pdf]

University of Glasgow | College of Medical, Veterinary & Life Sciences

Title of Project: Exploring faculty experiences and perceptions of interprofessional co-debriefing practice in healthcare simulation: A qualitative study

Introductions, thanking participants for attending, checking participants have had the opportunity to read the Participant Information Sheet and privacy notice, answer any questions they may have, check consent and that participant is happy to proceed prior to beginning interview. Check recording is working.

Semi-Structured Interview Topic Guide

1. Can you describe your healthcare professional background and experience?

2. What are your current roles and responsibilities as a simulation-based educator?

3. Can you describe your experience, journey and development as a simulation-based educator?

4. What are your experiences of interprofessional co-debriefing? Do you believe it an effective technique? If so, why?

5. Have you experienced any challenges when participating in interprofessional co-debriefing? What do you think are the factors that may have influenced these challenges arising? How might these differ to when you have practiced single faculty debriefings?

6. How do you usually identify yourself professionally? Does this change depending on the context? Why do you think that may or may not be?

7. How do you feel your professional background affects your debriefing practice, specifically in an interprofessional co-debriefing context? If so, how?

8. Do you feel your professional background and identity affects how you're perceived in the debriefing settings, either by fellow faculty or participants? Do you think the people's professional identity impacts interactions between co-debriefers? If so, how so?

9. Are the interactions with interprofessional colleagues in a debriefing environment similar or dissimilar to interactions with similar colleagues in clinical settings?

10. There is a lot of literature examining power imbalances and hierarchy affecting interprofessional working in the clinical environment. Is this something you are aware of or have experience of in the context of interprofessional co-debriefing? Do you find power imbalances and hierarchy influence your practice in such environments? If so, how so? If not, why might this be?

11. Can you recall any instances of hierarchy becoming apparent during co-debriefing? If so, what happened? In your opinion, what contributes to the presence or absence of hierarchy in these settings?

12. Do you find power imbalances and hierarchy influence your practice when co-debriefing with a colleague from a different professional background? If so, how so? If not, why not?

13. How do you and your co-debriefers navigate differences in seniority, hierarchy or professional authority? Are there any strategies you use?

14. Have you experienced any negative effects of interprofessional co-debriefing? If so, what may have led them occurring? On reflection, do you think they may have impacted the learners or just you?

15. Is there anything else you would like to add that we've not covered?

16. Do you have any questions for me about this research or the next steps?

Close interview with thanks, and confirming use of recording, and reiterating ability for participant to withdraw from study.
