# Supplementary material for: Exploring faculty experiences and perceptions of interprofessional co-debriefing practice in healthcare simulation: a qualitative study protocol
Source: BMJ Open. 2025 Oct 21;15(10):e109231. doi: 10.1136/bmjopen-2025-109231 (PMC12548587; doi:10.1136/bmjopen-2025-109231)
Supplement: online supplemental file 2 [file bmjopen-15-10-s002.pdf]

University of Glasgow | College of Medical, Veterinary & Life Sciences

PARTICIPANT INFORMATION SHEET

1. Study title

Exploring faculty experiences and perceptions of interprofessional co-debriefing practice in healthcare simulation: A qualitative study

2. Invitation to participate in this research study

You are being invited to take part in a research study. Before you decide, it is important for you to understand why the research is being done and what it will involve. Please take time to read the following information carefully and discuss it with others if you wish. Please ask us if there is anything that is not clear or if you would like more information. If you decide to take part in this study, you will be given a copy of this Participant Information Sheet, the privacy notice and the consent form to keep.

3. What is the purpose of the study?

The aim of this study is to explore the perceptions and experiences of interprofessional co-debriefers in simulation-based education. By conducting interviews with simulation-based educators who practice interprofessional co-debriefing, we hope to gather valuable insights into this topic, which may impact practice in this field moving forward.

4. Why have I been invited to participate?

You have been invited to participate in this study because you are a simulation-based educator with experience of practicing interprofessional co-debriefing. Due to these experiences, your insights are of importance to this research.

5. Do I have to take part?

No, participation in this study is entirely voluntary- it is up to you to decide whether or not to take part. If you do decide to take part, you will be given this information sheet, privacy notice and consent form to keep and be asked to return an electronically signed consent form to us prior to the interview. If you decide to take part, you are still free to withdraw at any time and without giving a reason. What happens to your data in the event of withdrawal is detailed in section 10 of this Participant Information Sheet.

6. **What will participation involve?**

If you agree to participate, you will be asked to take part in a semi-structured interview conducted by one of the members of the research team. The interview will last approximately 45 minutes and will be conducted on the online platform Microsoft Teams. The interview will be conducted at a mutually convenient time. We would ask that you are not in a public place during the interview. The interviews will be recorded for accuracy, and the recordings will be transcribed for analysis. Once the interviews are transcribed, the audio-visual recordings of the interview will be securely deleted.

7. **What are the possible disadvantages and risks of taking part?**

There are no significant risks associated with participating in this study. However, participating in these interviews will take approximately 45 minutes, and unfortunately, we are unable to provide any financial compensation for your time.

8. **What are the possible benefits of taking part?**

Beyond personal reflection on your interprofessional co-debriefing practice, you will receive no direct benefit from taking part in this study. The information that is collected during this study may help us better understand complex interprofessional dynamics, so that we can improve interprofessional co-debriefing practice.

9. **Will my taking part in this study be kept confidential?**

Yes. Your confidentiality is of upmost importance to us. No one outside of the research team will know that you have taken part in this study. Please note that assurances on confidentiality will be strictly adhered to, excepting cases in which there are disclosures of serious professional misconduct that compromise patient safety, violate professional standards or breach legal obligations. In such cases, the research team may be obliged to contact relevant institutional or regulatory bodies or agencies.

10. **What will happen to my data?**

This study is compliant with General Data Protection Regulation (2018). All information collected during the course of this research will remain strictly confidential. All data will be stored securely in an encrypted password-protected folder within the University of Glasgow's secure Microsoft OneDrive for Business platform to which only the research team will have access. Furthermore, once the audio-visual recording of your interview has been transcribed and anonymised, the recording will be deleted. The anonymised transcriptions will continue to be stored in the encrypted password-protected folder within the University of Glasgow's secure Microsoft OneDrive for Business platform for a period of ten years. Your name, signed consent form and contact details will also be stored in a separate encrypted password-protected folder within the University of Glasgow's secure Microsoft OneDrive for Business platform for a period of ten years. This will not be linked to your anonymised interview data. After a period of ten years, all data will be securely deleted, unless its use for

future research purposes has been identified by the research team. In such cases, your anonymised data may be used in this fashion, but only with your explicit consent, and would be stored until completion of any such research project.

If you withdraw from the study prior to transcription and anonymisation of the audio-visual recording of your interview, you have the right to request for the data to be corrected or completely removed. However, once the audio-visual recordings have been transcribed and anonymised, we are unable to remove the data from the study.

11. What will happen to the results of the research study?

The findings of this study may be published in academic journals and presented at scientific conferences. This may include direct quotations- in such cases the quotations will remain anonymised, ensuring you cannot be identified. If you wish to receive a summary of the research findings, this can be provided to you upon request.

12. Who is organising and funding the research?

This study is being led by the Principal Investigator, Dr Prashant Kumar (Department of Anaesthesia, NHS Greater Glasgow & Clyde & University of Glasgow). It is being part-funded by a research grant from the Scottish Society of Anaesthetists. Other sources of funding for dissemination of this work are also being explored.

13. Who has reviewed the study?

This study has been reviewed and approved by the University of Glasgow College of Medical, Veterinary & Life Sciences Ethics Committee.

14. Contact for Further Information

If you have any questions or concerns regarding this study or your participation, please contact any member of the research team:

Dr Prashant Kumar (Principal Investigator): Prashant.kumar@glasgow.ac.uk //
Prashant.kumar2@nhs.scot

Dr Susan Somerville: s.g.somerville@dundee.ac.uk

Thank you for taking the time to read this participant information sheet. Please do not hesitate to contact us if you have any further questions or if you wish to participate.
