# Supplementary material for: Exploring faculty experiences and perceptions of interprofessional co-debriefing practice in healthcare simulation: a qualitative study protocol
Source: BMJ Open. 2025 Oct 21;15(10):e109231. doi: 10.1136/bmjopen-2025-109231 (PMC12548587; doi:10.1136/bmjopen-2025-109231)
Supplement: online supplemental file 3 [file bmjopen-15-10-s003.pdf]

Privacy Notice for the following Research Project: Exploring faculty experiences and perceptions of interprofessional co-debriefing practice in healthcare simulation: A qualitative study

Your Personal Data

The University of Glasgow will be what's known as the 'Data Controller' of your personal data processed in relation to your participation in the above study. This privacy notice will explain how The University of Glasgow will process your personal data.

Why we need it

We are collecting your basic personal data such as your name and email, in case we need to contact you following your interview. Limited demographic data such as age range, gender, regional location (Scotland, Northern Ireland, England, Wales), healthcare professional background (e.g. nursing, medical), and years of interprofessional co-debriefing experience will be collected at the time of the interview and inserted into anonymised transcripts of your interviews. Once anonymous transcriptions of your interview have been completed, the video recordings will be securely deleted and there will no way to identify and link these data with your name or identity.

Legal basis for processing your data

We must have a legal basis for processing all personal data. In this instance, the legal basis is for the performance of a task in the public interest.

What we do with it and who we share it with

All the personal data you submit is processed by members of the research team, with the principal investigator being based at the University of Glasgow in the United Kingdom. Your personal data will not be shared with any third parties, either within or outside of the UK. Only members of the research team will have access to your data. Your data will be securely stored in a folder within an encrypted password-protected OneDrive. Transcriptions of your interviews will remain anonymised throughout the storage.

How long do we keep it for

Your data will be retained by the University for a period of ten years in a folder within a secure encrypted password-protected OneDrive. After this time, your data will be securely deleted, unless its use for future research purposes has been identified by the research team. In such a case, your explicit permission will be sought.

What are your rights?*

You can request access to the information we process about you at any time. If at any point you believe that the information we process relating to you is incorrect, you can request to see this information and may in some instances request to have it restricted, corrected or erased. If you withdraw from the study prior to transcription and anonymisation of the audio-visual recording of your interview, you have the right to request for the data to be corrected or completely removed. However, once the audio-visual recordings have been transcribed and anonymised, we are unable to remove the data from the study.

If you wish to exercise any of these rights, please submit your request via the webform or contact dp@gla.ac.uk.

*Please note that the ability to exercise these rights will vary and depend on the legal basis on which the processing is being carried out.

Complaints

If you wish to raise a complaint on how we have handled your personal data, you can contact the University Data Protection Officer who will investigate the matter. Our Data Protection Officer can be contacted at dataprotectionofficer@glasgow.ac.uk

If you are not satisfied with our response or believe we are not processing your personal data in accordance with the law, you can complain to the Information Commissioner's Office (ICO) https://ico.org.uk/
