# Supplementary material for: Exploring faculty experiences and perceptions of interprofessional co-debriefing practice in healthcare simulation: a qualitative study protocol
Source: BMJ Open. 2025 Oct 21;15(10):e109231. doi: 10.1136/bmjopen-2025-109231 (PMC12548587; doi:10.1136/bmjopen-2025-109231)
Supplement: online supplemental file 4 [file bmjopen-15-10-s004.pdf]

University of Glasgow | College of Medical, Veterinary & Life Sciences

**Title of Project:** Exploring faculty experiences and perceptions of interprofessional co-debriefing practice in healthcare simulation: A qualitative study

# CONSENT FORM

Please read the following statements and return a signed form to the study investigators. You will be asked for your verbal agreement with these statements at the beginning of the interview to confirm that you have consented to participate in the study.

Please initial box

- I confirm that I have read and understood the Participant Information Sheet Version 1 dated March 2025. ☐
- I confirm that I have read and understood the Privacy Notice Version 1 dated March 2025. ☐
- I have had the opportunity to think about the information, ask questions, and understand the answers I have been given. ☐
- I understand that my participation is voluntary and that I am free to withdraw at any time, without giving any reason, without my legal rights being affected. ☐
- I agree to my interview being audio-visually recorded and understand that the recording will be transcribed word by word, and that data will be stored for up to 10 years in accordance with relevant Data Protection policies and regulations. ☐
- I understand that my information and things that I say in an interview may be quoted in reports and articles that are published about the study and presented at scientific meetings, but that my name or any other identifiable information that could tell people who I am will not be revealed. ☐
- I understand that all data and information I provide will be kept confidential and will be seen only by study researchers and regulators whose job it is to check the work of researchers (excepting cases of serious professional misconduct). ☐
- I agree that my name, contact details, signed consent form and data described in the information sheet will be kept for a period of 10 years. ☐
- I understand that anonymised data from my interview may be used for future research purposes identified by the research team. I understand that in such a case, I would be asked to provide further consent, but that I would be under no obligation to give consent. ☐

- I understand that if I withdraw from the study after the audio-visual recording of my interview has been transcribed and anonymised, my data collected up to that point may be retained and used for the remainder of the study. ☐

- I agree to take part in the study. ☐

| | | |
|---|---|---|
| ______________________ | __________ | ______________________ |
| Name of participant | Date | Signature |
| ______________________ | __________ | ______________________ |
| Name of Person taking consent (if different from researcher) | Date | Signature |
| ______________________ | __________ | ______________________ |
| Researcher | Date | Signature |

(1 copy for participant; 1 copy for researcher)
