# Supplementary material for: Exploring faculty experiences and perceptions of interprofessional co-debriefing practice in healthcare simulation: a qualitative study protocol
Source: BMJ Open. 2025 Oct 21;15(10):e109231. doi: 10.1136/bmjopen-2025-109231 (PMC12548587; doi:10.1136/bmjopen-2025-109231)
Supplement: Supplementary file 5 [file bmjopen-2025-109231.reviewer_comments.pdf]

PEER REVIEW HISTORY

BMJ Open publishes all reviews undertaken for accepted manuscripts. Reviewers are asked to complete a checklist review form and are provided with free text boxes to elaborate on their assessment. These free text comments are reproduced below.

ARTICLE DETAILS

Title (Provisional)

Exploring Faculty Experiences and Perceptions of Interprofessional Co-Debriefing Practice in Healthcare Simulation: A Qualitative Study Protocol

Authors

Kumar, Prashant; Groom, Olivia; Paterson, Stephen; McGowan, Neil; Allan, Russell; Sharp, Kathryn; Somerville, Susan

---

VERSION 1 - REVIEW

**Reviewer**	**1**
**Name**	**Sonden, Anders**
**Affiliation**	**Karolinska Institute, Dept of Clinical Science and Education**
**Date**	**10-Sep-2025**
**COI**	**None**

I find this a well-written protocol addressing an underexplored area and the proposed study is likely to generate valuable insights. The protocol is clear and methodologically sound. I particularly appreciate the authors' use of both constructivist and constructionist lenses.

That said, the manuscript would be further strengthened by the following clarifications and refinements:

1. The abstract is well-structured but somewhat generic. It could be sharpened by stating more clearly what unique gap in the literature this study addresses.

2. The authors state that "up to 30 participants" will be recruited. It would be useful to explain more explicitly why this number is considered adequate, in relation to expected data richness and variation.

3. The manuscript notes the risk of overrepresentation from certain professional groups. Could the authors elaborate on how they will actively seek diversity (e.g., profession, age, region, gender) to strengthen the breadth of perspectives?

4. While the topic guide is comprehensive, some questions (e.g., those focusing on hierarchy and power) may risk steering participants. Please clarify how the team will balance open

exploration with these prompts, and how potential bias from leading questions will be mitigated.

5. The study is limited to the UK. The authors acknowledge this, but it would strengthen the manuscript to discuss briefly how the findings may (or may not) translate to international settings with different professional hierarchies and simulation practices.

6. The section on data handling is detailed and appropriate. Please clarify whether anonymised data might be made available for secondary use by other researchers, or whether access will be restricted to the study team only

In conclusion, this is a strong and promising study protocol. Addressing the comments above, particularly around sampling and transferability, will make it even better.

VERSION 1 - AUTHOR RESPONSE

Reviewer 1's Comments	Authors' Comments / Changes / Justification
I find this a well-written protocol addressing an underexplored area and the proposed study is likely to generate valuable insights. The protocol is clear and methodologically sound. I particularly appreciate the authors' use of both constructivist and constructionist lenses	Thank you for your kind words. We're delighted that you enjoyed reading the manuscript and find it relevant and valuable.
1. The abstract is well-structured but somewhat generic. It could be sharpened by stating more clearly what unique gap in the literature this study addresses.	We have adjusted the abstract accordingly. We hope that you agree it is now more focussed and sharper. We have been clearer in highlighting the unique gap in the literature that this study is addressing.
2. The authors state that "up to 30 participants" will be recruited. It would be useful to explain more explicitly why this number is considered adequate, in relation to expected data richness and variation.	We have expanded the study design section to explain our rationale and referencing the information power model we are adhering to. We emphasise that 30 is a pragmatic upper limit to ensure data richness and depth while permitting broad coverage across professional groups, with adequacy judged iteratively using the information power model, and recruitment ceasing when sufficient information power is achieved or the maximum is reached.
3. The manuscript notes the risk of overrepresentation from certain professional groups. Could the authors elaborate on how they will actively seek diversity (e.g., profession, age, region, gender) to strengthen the breadth of perspectives?	We have elaborated on our sampling strategy in the "participants- recruitment and sampling strategy" section to highlight how our strategy actively seeks diversity in perspectives, across professional groups, background, debriefing experience and geographical regions. We have also referenced the strategy to signpost readers to the appropriate literature.
4. While the topic guide is comprehensive, some questions (e.g., those focusing on hierarchy and power) may risk steering participants. Please clarify how the team will balance open exploration with these prompts, and how potential bias from leading questions will be mitigated.	Thank you for these insights, and we acknowledge this concern. As a research team, we have reflected upon your comments about how best we can balance open exploration whilst mitigating the risk of bias from leading questions. The topic guide intentionally includes prompts on hierarchy and power, however we acknowledge the need to iteratively revise and refine the guide to ensure that prompts are open-ended and ensuing conversations participant-led. Such process of continuous revision is standard in reflexive qualitative research, where topic guides are expected to evolve to maintain openness and sensitivity to participants' framing of experience. We have therefore stated this intention within the manuscript in the 'study design' section. However, we also feel that it remains prudent to include the topic guide in its original form, as a

	supplementary file, as this is the initial framework used at the outset of data collection. This is to ensure full transparency to the reader thus allowing them to draw their own conclusions regarding impact on bias. On completion of the study, we will include multiple versions of the topic guide in ensuing publications to showcase the iterative revisions that it will likely go through. Furthermore, we have decided to undertake individual and group reflexive memos and group debrief discussions to monitor whether questions inadvertently prime responses and will include this information in the final reports. We have alluded to this strategy in the protocol manuscript in the 'study design' section.
5. The study is limited to the UK. The authors acknowledge this, but it would strengthen the manuscript to discuss briefly how the findings may (or may not) translate to international settings with different professional hierarchies and simulation practices.	We have added the following statement within the manuscript describing the limitations of a UK-centred study, and the implication this has for transferability of findings:<br><br>"We acknowledge that the findings from this study will be UK-centred. As such, the sociocultural dynamics of interprofessional co-debriefing within this context, specifically in respect to professional status, hierarchy and power, may not be directly transferrable to other international settings, where different sociocultural norms and simulation practices exist. Our aim, therefore, is not to afford judgment on transferability, but to transparently provide sufficiently detailed descriptions of context and analytic interpretations, such that readers can form their own judgements regarding transferability to their local settings."
6. The section on data handling is detailed and appropriate. Please clarify whether anonymised data might be made available for secondary use by other researchers, or whether access will be restricted to the study team only	We have added a statement within the data handling section to clarify that access to all anonymised data is restricted to the study team only, and is unavailable for secondary use by other researchers (as per the participant consent process).
In conclusion, this is a strong and promising study protocol. Addressing the comments above, particularly around sampling and transferability, will make it even better.	Thank you for your comments and suggestions. We hope you agree that, with the alterations we have made in response to your recommendations, the manuscript is now much stronger and suitable for publication. Thank you for the time and diligence you have taken in reviewing our work, it is much appreciated.

VERSION 2 - REVIEW

Reviewer 1

Name Sonden, Anders

Affiliation Karolinska Institute, Dept of Clinical Science and Education

Date 18-Sep-2025

COI

The authors have addressed my suggestions for revision in a very satisfactory manner. I recommend the protocol for publication and look forward to reading the forthcoming article.
